# New Insights on Y, La, Nd, and Sm Extraction with Bifunctional Ionic Liquid Cyphos IL 104 Incorporated in a Polymer Inclusion Membrane

**DOI:** 10.3390/membranes14090182

**Published:** 2024-08-23

**Authors:** Mohamed Malki, Lynda Mitiche, Amar Sahmoune, Clàudia Fontàs

**Affiliations:** 1 Laboratory of Physics and Materials Chemistry (LPCM), University Mouloud Mammeri of Tizi Ouzou, Tizi Ouzou 15000, Algeria; mohamed.malki@ummto.dz (M.M.); lynda.mitiche@ummto.dz (L.M.); amar.sahmoune@ummto.dz (A.S.); 2 Department of Chemistry, University of Girona, C/Maria Aurèlia Capmany 69, 17003 Girona, Spain

**Keywords:** rare earth elements (REEs), polymer inclusion membranes (PIM), bifunctional ionic liquid extractant, Cyphos IL 104, separation

## Abstract

In this study, an ionic liquid-based polymer inclusion membrane (IL-PIM) made of (50% polymer-50% CyphosIL104) was used to extract and separate the rare earth elements (REEs) Y, La, Nd, and Sm in chloride solutions. The effect of extraction time and pH was studied to optimize the extraction and separation conditions. The four REEs were effectively extracted at pH 4–5 from both single and mixed metals solutions. However, at pH 2, only Y was extracted. The recovery of the extracted REEs from the loaded PIM was achieved using HNO_3_ and H_2_SO_4_. In the case of La, it was quantitatively back-extracted with H_2_SO_4_ after a contact time of 1 h, while up to 4 h was necessary to recover 70% of the extracted Y, Sm, and Nd. Extraction isotherms were studied, and the Freundlich isotherm model was the most adequate to describe the interaction between the PIM and the REEs. Finally, the developed PIM was investigated for the extraction of REEs from mixtures containing other metals, which showed great selectivity for the REEs.

## 1. Introduction

Rare earth elements (REEs) are generally found together in the same minerals as phosphates, oxides, carbonates, silicates, and halides with some non-metals but are also present in trace amounts in the ores of other metals as co-products or by-products [1,2,3,4]. REEs are the most used metals in many innovative technologies, including those related to green energy, such as photovoltaic devices, lithium-ion batteries, GPS geolocation screens, catalysts, and permanent magnets, as well as metallurgy. Examples of the use of rare earth include the production of miniaturized permanent magnets, which are widely used in electric vehicle motors and wind turbines (Nd, Sm, Pr, and Dy), battery alloys (La), optical fiber and lasers (Er), screen phosphors (Eu), and in metallurgy and ceramics (Y) [5,6,7,8]. The availability of rare earths in natural deposits has been greatly reduced by these extensive uses [9]. Furthermore, the fact that rare earths have identical physico-chemical properties and small differences in their ionic radius make their industrial chemical separation both more difficult and more costly [10].

In order to overcome these problems, efficient and environmentally friendly methods need to be developed for their separation and recovery from secondary sources such as metal and non-metal ore [11]. Solvent extraction (SX) is one of the most efficient methods used for the separation and industrial production of rare earths [12,13,14]. Although this method can produce rare earths of high purity, multiple extractions are required for this to be achieved. Large amounts of toxic chemical products need to be used, rendering it economically and environmentally unsustainable.

Other conventional methods have also been employed to recover rare earth, including adsorption [15], ion exchange [16], and crystallization by precipitation [17]. However, these technologies produce secondary sludge and are less efficient in recovery and separation at low concentrations. Functionalized membranes, such as those containing specific extractants (carriers), including bulk-liquid membranes (BLMs) [18], emulsion liquid membranes (ELMs) [19,20], supported liquid membranes (SLMs) [21,22,23], and polymer inclusion membranes (PIMs) [24,25,26] have all been successfully employed to separate rare earths from different sources. They each offer distinct advantages, including easy preparation, low consumption of extracting carriers, high transport flux, and fast permeation [27,28,29,30].

Among these, PIMs are the most stable functionalized membranes since the carrier is entrapped in a polymeric matrix, avoiding leakage. The most commonly used carriers in PIMs to separate REEs are di-(2-ethyl hexyl) phosphoric acid (D2EHPA), trioctylphosphine oxide (TOPO), and tributyl phosphate (TBP), as solvating extractants [19,21,25,31,32], and quaternary ammonium salts [33]. Although these extractants are highly effective for metal extraction, some of them are not suitable due to their toxicity and harmful effects on the environment.

More recently, ionic liquids (ILs), which are thermally stable, not volatile, and less toxic reagents, have been employed as a good alternative [34,35]. Moreover, ILs incorporated in PIMs have the particularity of acting not only as the agent responsible for extracting the metal but also as a plasticizer, resulting in mechanically stable films. It has been reported that the immobilization of an IL in the polymer increases its stability and, in some cases, enhances the rate of transport and diffusion of the extracted species [36,37]. The phosphonium-based IL trihexyl(tetradecyl)phosphonium bis(2,4,4-trimethylpentyl)phosphinate (Cyphos IL104) is a bifunctional ionic liquid extractant since both cation and anion are involved in the extraction. This extractant demonstrated exceptional efficacy in removing or recovering low concentrations of metal ions from aqueous solutions without causing secondary contamination. This underscores the significant advantages of this ionic liquid in the recovery of rare earth elements [38,39,40,41,42].

The extractants Cyphos IL104 and bis(2,2,4-trimethylpentyl)phosphonic acid (Cyanex 272) were used for the solvent extraction of La, Nd, Gd, and Lu from single and mixed chloride solutions, showing Cyphos IL 104 to be the best extractant, although Cyanex 272 exhibited better selectivity toward heavier trivalent lanthanides [43]. Comparative studies between Cyphos IL 104 and D2EHPA and ionic liquid [Aliquat 336/Cyanex 272] on europium(III) and yttrium(III) extraction from single and mixed chloride media have also been undertaken. The results showed that D2EHPA was more suitable for Y(III)/Eu(III) separation, but the selectivity of Eu(III)/La(III) and Eu(III)/Ce(III) was better with Cyphos IL 104 [38]. However, higher extraction abilities for Y(III) were observed using the two ionic liquids [Aliquat 336/Cyanex 272] and Cyphos IL 104 from chloride and nitrate media [44].

Cyphos IL 104 has also been successfully incorporated in a PIM made of cellulose triacetate (CTA) as the base polymer and 2-nitrophenyl octyl ether (NPOE) as a plasticizer. This PIM was investigated for the competitive and selective transport of La(III) and Ce(III) from aqueous nitrate solutions containing various metal ions [39]. Similarly, a PIM with the same extractant was used for the separation of lutetium (III) and ytterbium (III) [40]. However, the extraction of rare earths by the PIM made of CTA and Cyphos IL 104 without a plasticizer has not been reported.

The current study reports the use of a functionalized membrane made of CTA and Cyphos IL 104 for the solid-liquid extraction and separation of yttrium, neodymium, samarium, and lanthanum from both single aqueous solutions and mixtures. Parameters affecting the extraction system, such as pH and extraction time, have been evaluated, as well as the back-extraction of the REEs, which was achieved using mineral acids. Furthermore, Freundlich and Langmuir’s isotherm models have been applied to the equilibrium extraction data to gain insight into the behavior of the PIM as an REE sorbent.

This study presents a simple extraction methodology that offers a new material that is easy to prepare and handle, which enables the effective recovery of these valuable elements.

## 2. Materials and Methods

### 2.1. Reagents and Solutions

Hydrochloric acid (HCl), sulphuric acid (H_2_SO_4_), nitric acid (HNO_3_), acetic acid/sodium acetate buffer (CH_3_COOH/CH_3_COONa), and sodium chloride salt (NaCl), all of the analytical grade, were purchased from PanReac AppliChem (Barcelona, Spain). Cyphos IL104 was purchased from Sigma-Aldrich (Darmstadt, Germany). Cellulose Triacetate (CTA) was purchased from Acros Organics (New Jersey, USA).

Chloroform, used to dissolve and mix CTA and Cyphos IL104, was purchased from Sigma Aldrich (St Louis, MI, USA). La(III) and Sm(III) stock solutions were prepared by dilution from their element reference solutions of a concentration of 1000 mg L^−1^ and were purchased from ROMIL Pure Chemistry (Barcelona, Spain). Nd(III) and Y(III) stock solutions were prepared by dilution from their standard solutions for inductively coupled plasma (ICP) that have a concentration of 1000 mg L^−1^ and were purchased from Sigma-Aldrich (Buchs, Switzerland).

Solutions for the extraction studies were prepared containing 10 mg L^−1^ of each individual metal at 0.05 M NaCl and pH = 5 (using acetate buffer). For competitive extraction studies, a solution containing a mixture of 10 mg L^−1^ of each metal in the same media was used. The choice of the salting-out agent and its concentration is based on studies already reported in the literature [43,45].

### 2.2. Polymer Inclusion Membrane Preparation

PIMs were prepared following the procedure already described by Sugiura et al. [24] using 200 mg CTA and the same mass of Cyphos IL104, dissolved in 20 mL chloroform and using a 9.0 cm diameter flat-bottom glass Petri dish. After the evaporation of chloroform, the film was carefully peeled off the bottom of the Petri dish. The resulting membrane had a mean thickness of 55 ± 5 µm. For the extraction experiments, pieces of 2 cm × 2 cm were cut (mass = 0.025 ± 0.005 g). From each PIM, 10–12 pieces were obtained.

The structure of PIM components and their properties are reported in Table 1.

### 2.3. Extraction Experiments

Extraction experiments were performed by contacting 10 mL of 10 mg L^−1^ individual metals in 0.05 M NaCl and pH 5 with the 4 cm^2^ membranes described above, usually during 24 h under orbital shaking. All the extraction experiments were carried out in triplicate. The extraction efficiency (%) was calculated as following in Equation (1):

(1) $$Extraction\;\left(\%\right)=\frac{Ci-Ct}{Ci}\times 100,$$

*C_i_* is the initial concentration of the REEs, and *Ct* is the concentration of the REEs after the extraction experiment at a given time.

### 2.4. Back-Extraction of Rare Earths

REE back-extraction was investigated using 1 M HNO_3_ and 1 M H_2_SO_4_ as stripping agents [46]. Experiments were carried out by mixing 10 mL of each acid solution with the loaded PIM under orbital shaking for a specific period of time.

Back-extraction efficiency was calculated as following in Equation (2):

(2) $$Back-extraction\;\left(\%\right)=\frac{Ce}{Ci-Ce}\times 100,$$

where *C_e_* is the REE equilibrium concentration in the aqueous solution after back-extraction.

### 2.5. Separation Factor

For the separation of REE from heavy metal mixtures, the SF was calculated as following:

(3) $${SF}_{REE/M}=\frac{D_{REE}}{D_{M}}$$

where *D_REE_* represents the distribution rate of the REE, and *D_M_* the distribution ratio of the metals (M) Cu, Co, Ni, and B, used in competitive studies (Section 3.3).

The distribution rate of both REE and M is calculated as follows:

(4) $$D=\frac{Ci-Ce}{Ce}$$

where *C_i_* and *C_e_* represent the initial concentration, and the equilibrium concentration of the REE or M, respectively.

### 2.6. Freundlich and Langmuir Isotherm

The results of extraction according to the Freundlich and Langmuir kinetic models are expressed taking into account variable initial yttrium concentrations (2–20 mg L^−1^) with fixed PIM mass (0.025 ± 0.005 g).

The Freundlich isotherm:

*q_e_* = *K_F_ C_e_^n^* (5)

where *q_e_* represents the equilibrium sorption capacity of sorbent in mg (metal) per g (adsorbent), and *C_e_* is the equilibrium concentration (mg L^−^^1^). *K_F_* and n are constants. *q_e_* can be calculated as follows:

*q_e_* _=_ (*C_o_* − *C_e_*) *V*/*m_ads_* (6)

*C_o_* is the initial concentration of metal ions (mg L^−^^1^), and *V* is the volume of solutions (mL).

The linear form Freundlich isotherm can be written as follows:

*Log q_e_* = *log K_F_* + *n log C_e_* (7)

The Langmuir isotherm:

*q_e_* = *K_L_q_m_ C_e_*/(1 + *K_L_ C_e_*) (8)

*q_m_*: maximal constant of sorption of metal per unit mass of sorbent in forming a complete monolayer on the surface. *K_L_*: equilibrium constant of the Langmuir isotherm. *m_ads_*: the mass of the adsorbent (g).

A linear regression can be performed to obtain the constants *q_m_* and *K_L_* if the model is validated as follows:

(*C_e_*/*q_e_*) = (1/*K_L_q_m_*) + (1/*q_m_*) *C_e_* (9)

### 2.7. Apparatus

The characterization of the PIM morphology was investigated by a digital scanning electron microscope (SEM) with a field emission electron source (FE-SEM) Zeiss, model DSM 960 (Oberkochen, Germany), with a resolution of 30–2 µm. Information on the membrane functional groups was recorded by infrared spectra using an Agilent Cary 630 FTIR spectrometer. Thermal characterization of the membrane consisted of thermogravimetric analysis (TGA) by a Mettler Toledo TGA/DSC combined instrument with a temperature range from 30 °C to 1600 °C. The thickness of the membranes was measured using a Digimatic Micrometer 0–25 mm, Mitutoyo (Tajatsu-ku, Japan). A microwave plasma-atomic emission spectrometer (Agilent Technologies, Agilent 4200 MP-AES, Santa Clara, California, USA) was used to measure REE concentrations in the aqueous solutions. La at λ = 394.910 nm, Y at λ = 371.029 nm, Nd at λ = 430.358 nm, Sm at λ = 442.434 nm.

## 3. Results and Discussion

### 3.1. Membrane Characterization

**Figure 1:**
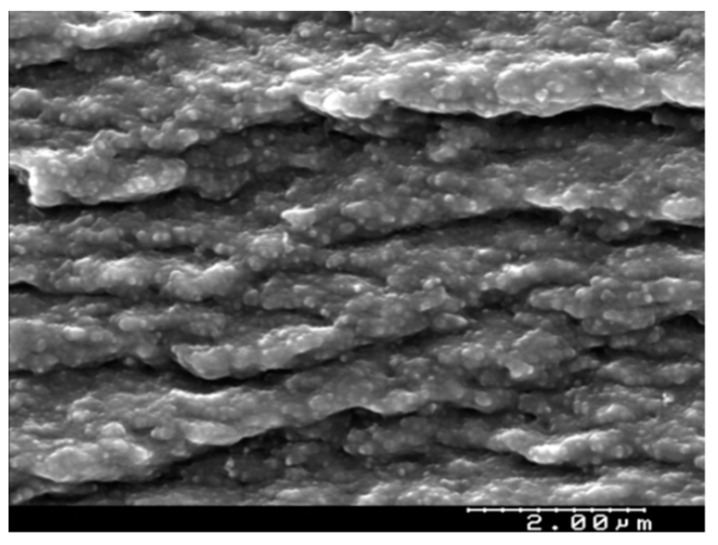
SEM micrographs of the cross section of the PIM composed of 50% CTA and 50% Cyphos 104 IL104.

Different techniques were used to obtain information about the characteristics of the PIM. Figure 1 shows the SEM of the cross-section of the PIM 50% CTA and 50% Cyphos IL104. As can be seen, the membrane is dense, with no pores, and presents a uniform layer. Similar images were obtained by Baczyńska et al., who studied the structure of membranes based on CTA with Cyphos IL 104 as a carrier [47].

**Figure 2:**
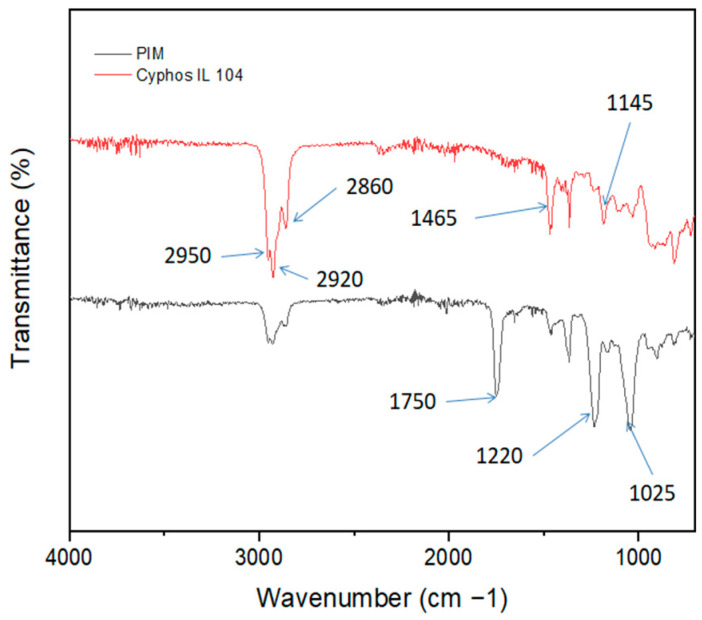
FT−IR spectra of Cyphos IL104 and PIM with a composition of 50%CTA and 50% Cyphos IL104.

Figure 2 displays the Fourier infrared transformed spectra of Cyphos IL 104 (in red) and the PIM (in black). The FT-IR spectra obtained show numerous absorption bands around 2950–2920 cm^−1^/2860 cm^−1^ that are specific to different functional groups of Cyphos IL 104 and CTA. The peaks at 2950 cm^−1^ correspond to the stretching vibrations of C-H bands of saturated-(CH_3_), while the peaks at 2920 cm^−1^ and 2860 cm^−1^ represent the stretching vibrations of –(CH_2_)- present in the PIM. The presence of characteristic peaks at the fingerprint region of both Cyphos IL 104 and PIM around 1465 cm^−1^, 1145 cm^−1^, and 1025 cm^−1^ correspond to the stretching vibration of P–C, P=O, and –P-O bands, respectively.

The peaks at 1750 cm^−1^ and 1220 cm^−1^ correspond to the stretching vibrations of the carbonyl group (C=O) and C–O single bands, respectively.

**Figure 3:**
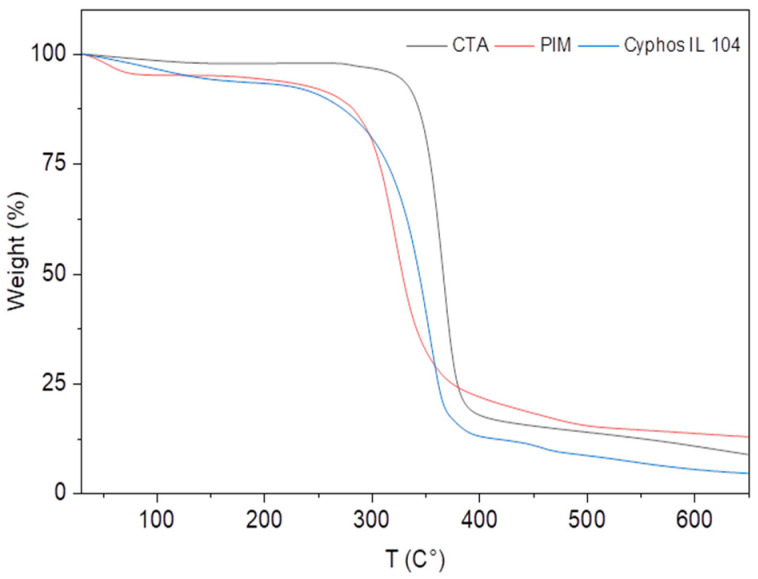
TGA of Cyphos IL104 (Blue), CTA (Black), and PIM (Red), mass Cyphos IL104 = 5.8 mg, mass CTA = 7.4 mg, m PIM = 6.1 mg.

Moreover, TGA analysis was carried out to assess the thermal stability and decomposition behavior of the membrane material. Figure 3 shows the results for the pure components CTA and Cyphos IL 104, as well as the PIM. The thermogram of pure CTA on heating from room temperature to 700 °C shows that there is no molecular degradation below 200 °C. Two stages of CTA degradation are observed above this temperature. The first stage, which appears at about 300 °C, represents the thermal degradation of the main chain of cellulose triacetate, and the second stage, which starts at 350 °C, represents the carbonization of the compound. For Cyphos IL 104, the evaporation of water molecules occurs first, followed by the degradation of the compound starting at 315 °C.

In the case of the PIM, TGA characterization indicates that at 300 °C, the intermolecular interactions between the various constituents of the PIM are weak, as evidenced by the fact that both the extractant and polymer decomposed at temperatures close to their boiling points [40].

### 3.2. Extraction Experiments

#### 3.2.1. Individual Rare Earth Extraction Studies

The extraction abilities of Cyphos IL 104, incorporated in a PIM, were first investigated for Y, Nd, Sm, and La from single aqueous solutions. Both the pH and contact time are among the most significant parameters that can influence REE separations and the uptake of metal ions. Therefore, the effect of pH was studied, and the extraction kinetics were performed by contacting 10 mL of each REE solution at a concentration of 10 mg L^−1^ in 0.05 M NaCl at both pH 2 and 5 for periods of 1 and 24 h.

**Figure 4:**
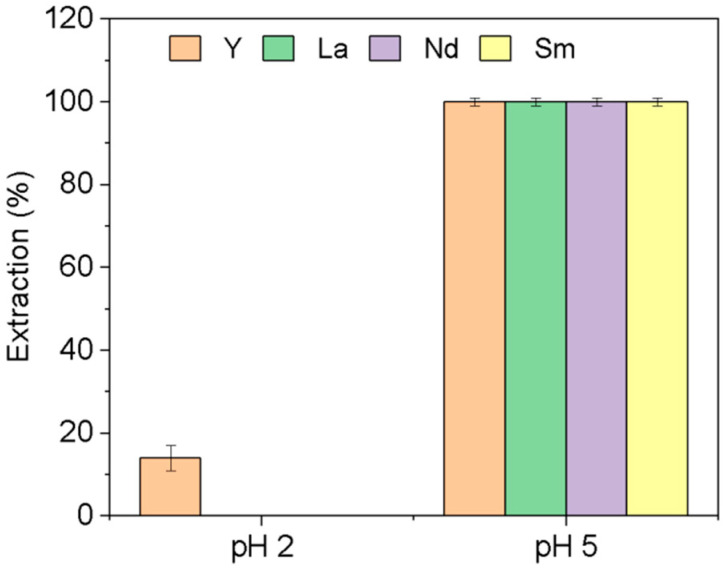
Extraction of individual rare earths at different pH. [REE] = 10 mg L^−1^, [NaCl] = 0.05 M, V = 10 mL, time = 1 h, (n = 3).

As can be seen in Figure 4, the extraction of the four REEs is pH dependent, and was quantitative at pH = 5 after contact time of only 1 h, while at pH 2, only Y is weakly extracted (14 ± 3%).

**Figure 5:**
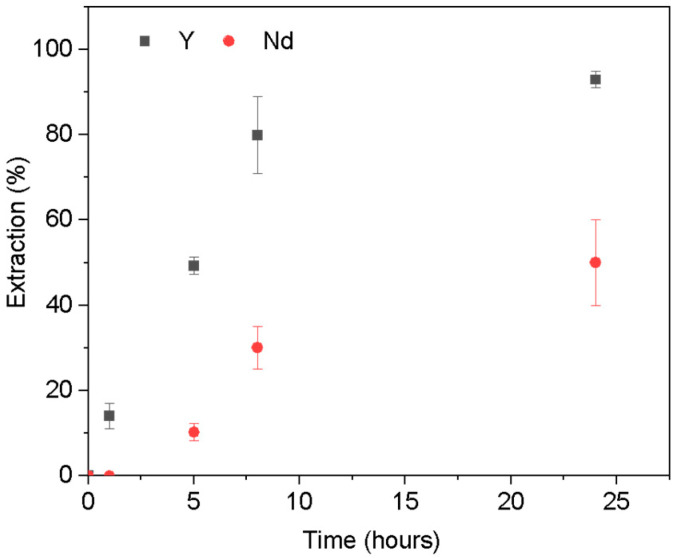
Extraction kinetics of Y (black) and Nd (red) at pH 2. [REE] = 10 mg L^−1^, [NaCl] = 0.05 M, V = 10 mL, (n = 3).

To better understand the extraction at pH 2, a kinetic study was conducted for Y and Nd with varying contact times from 15 min to 24 h. The results are presented in Figure 5. As depicted, the PIM exhibits a higher affinity for Y, which is extracted immediately, whereas Nd requires more time for extraction. Additionally, it is worth noting that the extraction process for Y continues to increase sharply within the first 8 h, ultimately reaching a maximum extraction efficiency of 93% after 24 h. Although the extraction rate for Nd is relatively low compared to Y, it gradually increases over time. This difference in extraction kinetics can be exploited to design efficient separation processes, allowing for the selective recovery of Y before Nd is significantly extracted at pH 2.

The mechanisms by which rare earths are extracted by bifunctional ionic liquids such as Cyphos IL 104 are poorly understood. Some authors have explained that the extraction of REE from chloride and nitrate medium with Cyphos IL 104 is accomplished by an anion-exchange mechanism [38,39,43,46], while others suggested that Cyphos IL 104 can be acidified as HNO_3_ -Cyphos IL 104 and its cation and anion involved in the extraction of REE via a coordinating mechanism [48]. In the case of an ion-exchange mechanism for REE extraction from a chloride medium, the extraction process can be described as follows:

(10) $$(REE{Cl}_{3}{)}_{Aq}+3(R_{3}R^{\prime }PA{)}_{Org}↔3(R_{3}R^{\prime }PCl{)}_{Org}+(REEA_{3}{)}_{Org}$$

where R_3_R′P and A are, respectively, the organic cation (trihexyl(tetradecyl) phosphonium), and the organic anion (bis(2,4,4-trimethylpentyl) phosphinate) of Cyphos IL104.

#### 3.2.2. Extraction Isotherms

**Figure 6:**
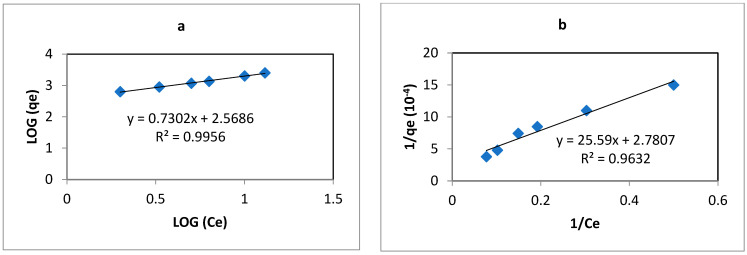
(**a**) Freundlich and (**b**) Langmuir linearized isotherms for Y(III), [Y] = 2–20 mg L^−1^, V = 10 mL, pH = 2, m PIM = 0.025 ± 0.005 g.

Freundlich and Langmuir isotherm models were used to analyze the interaction between the PIM and the REEs. Sorption (or extraction) isotherm experiments were conducted for yttrium to find the efficiency of the PIM with varying initial concentrations at 24 h contact. Figure 6a,b show the Freundlich and Langmuir isotherm curves in linearized form and the correlation coefficients deduced from Equations (8) and (9). The values of the correlation coefficient R^2^ ranging between 0.995 and 0.962 show that the Freundlich model best fits the experimental data compared to the Langmuir model, suggesting that the extraction mechanism of yttrium in the PIM was not onto a uniform site but via chemisorption by anion exchange with Cyphos IL 104. A higher value of parameters K_F_ (370.34) and Freundlich exponent (n) (0.7302) suggests favorable sorption conditions. The maximum sorption amount obtained was q_m_ = 2.91 (10^−5^ moles/g_PIM_).

#### 3.2.3. Back-Extraction of Individual REEs from Loaded PIM

To back-extract REEs from the loaded membrane, we tested the mineral acids HNO_3_ and H_2_SO_4_ at 1 M concentration, with contact times of 1 and 4 h. In Table 2, the results showed that H_2_SO_4_ has a higher ability to back-extract lanthanum than HNO_3_. However, similar results on the back-extraction efficiency were achieved for the other metals (i.e., Y, Nd, and Sm) using either HNO_3_ or H_2_SO_4_. In no case was the back-extraction efficiency of the REEs enhanced by increasing the contact time, with maximum back-extraction obtained after 1 h.

### 3.3. Competitive REE Extraction

**Figure 7:**
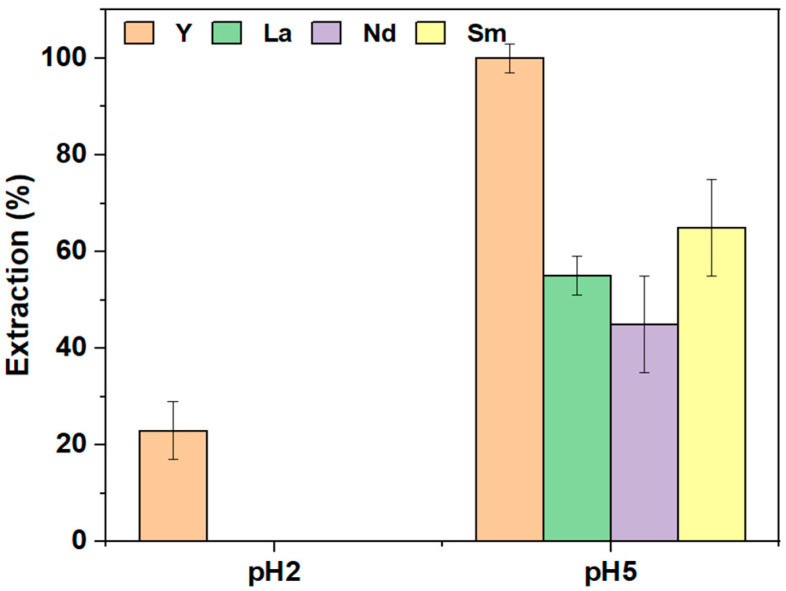
Selective extraction of REEs, Y (orange), La (green), Nd (violet), and Sm (yellow), [REE] = 10 mg L^−^^1^, V = 10 mL, [NaCl] = 0.05 M, pH = 5, time = 24 h (n = 3).

The extraction of REEs from mixtures containing Y, La, Nd, and Sm using the PIM was investigated at pH 2 and pH 5 over a 24-h extraction period. Competitive extraction results are shown in Figure 7. At pH 2, only Y was extracted, achieving a separation efficiency of 23 ± 6% from the other REEs, indicating promising selectivity, though the desired extraction efficiency was not reached. At pH 5, all elements were extracted, with Y being quantitatively extracted (100 ± 3%), followed by Sm (65 ± 10%), La (55 ± 4%), and Nd (45 ± 10%). This suggests that Cyphos IL 104 exhibits greater selectivity towards Y compared to Sm, La, and Nd, with selectivity increasing as the ionic radius of the elements decreases, consistent with the literature observations for chelating agents [49].

**Figure 8:**
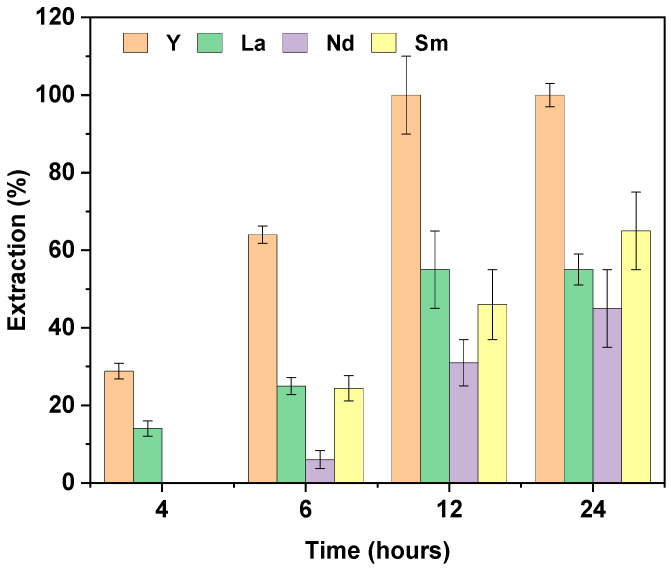
Effect of time on the extraction of REEs in a mixed solution. [REE] = 10 mg L^−^^1^, V = 10 mL [NaCl] = 0.05 M, pH = 5, m PIM = 0.025 ± 0.005 g (n = 3).

Since pH 5 allowed for the extraction of all four elements, we investigated the effect of contact time on the extraction of these elements from a mixture. Experiments were conducted at different times of agitation of 4, 6, 12, and 24 h, with the results shown in Figure 8. Initially, at shorter contact times (4 and 6 h), the extraction kinetics were slow, and the extraction efficiency was relatively low. Specifically, at 4 h, Y and La were selectively separated from Sm and Nd, indicating that shorter extraction times might favor the separation of these REEs, although the overall extraction quantities were limited. As the contact time increased to 12 h, higher extraction efficiencies were achieved. At this point, Y was preferentially extracted compared to the other REEs, with the extraction order being Y (100 ± 10%) > La (55 ± 10%) > Sm (46 ± 10%) > Nd (31 ± 6%). At 24 h, the extraction of Nd and Sm showed only a slight improvement, while the extraction of La and Y remained unchanged.

Given that the rare earth elements Y, Nd, Sm, and La may be present with other transition metals in both their ores and technological devices, we also investigated the extraction of each REE from mixed solutions containing competing B and Co(II), Ni(II) and Cu(II) metal ions. The different mixtures and concentrations were chosen to mimic the concentration ratios in powders produced from crushing end-of-life technological devices and permanent magnets for recycling, as has already been reported [50]. The results, summarised in Table 3, show that B, Co, Cu, and Ni are not extracted into the PIM. Nd was quantitatively extracted, while the extraction of Sm was 65 ± 5%.

## 4. Conclusions

In this study, we have demonstrated that a PIM containing Cyphos IL 104 effectively extracts the rare earth elements Y, La, Nd, and Sm and that the success of the extraction depends on the contact time and the pH of the aqueous solution. Effective extraction of all four elements was achieved at pH 5 in single-element solutions, whereas at pH 2, only Y was quantitatively extracted. In competitive extraction from REE mixtures, Cyphos IL104 exhibited higher selectivity for Y compared to Sm, La, and Nd, with selectivity increasing as the ionic radius decreased. Furthermore, in solutions with competing divalent metals, Nd was quantitatively extracted, and Sm was partially extracted at pH 5 without co-extraction of the divalent metals. The extraction of La was not affected by the presence of other metals. The results highlight the potential of this low-cost membrane material for efficient extraction of rare earth elements, which is highly relevant for both environmental and industrial applications.

## Figures and Tables

**Table 1:** Chemical structure and main properties of the PIM’s components.

Chemical Structure of PIM Components	Properties
Extractant: Cyphos IL 104 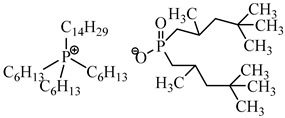 Polymer: CTA 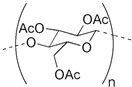	Molecular formula: Physical state 25 °C: Yellow viscous liquid Molar mass (g mol^−1^): 773.27 Density (g mL^−1^): 0.895 Viscosity at 25 °C: 806 mPa S T_m_ (melting temperature) = 302 °C

**Table 2:** Effect of time on REEs back-extraction from loaded PIM, stripping agents: 1 M H_2_SO_4_ and HNO_3_ acid, V = 10 mL, (n = 3).

Eluents	Time (h)	Back-Extraction Efficiency (%)			
		Y	La	Nd	Sm
HNO_3_	1	62 ± 6	72 ± 5	63 ± 10	66 ± 11
	4	70 ± 10	74 ± 5	75 ± 10	67 ± 7
H_2_SO_4_	1	64 ± 6	100	73 ± 11	62 ± 6
	4	64 ± 18	100	74 ± 3	61 ± 8

**Table 3:** Separation of REEs from heavy metal solutions, [REE] = 10 mg L^−^^1^. Solution 1: [Co] = 3 mg L^−^^1^, [B] = 1 mg L^−^^1^. Solution 2: [Co] = 20 mg L^−^^1^, [Cu] = 2 mg L^−^^1^. Solution 3 = [Co] = [Ni] = [Cu] = 10 mg L^−^^1^. All solutions: [NaCl] = 0.05 M, pH = 5, V = 10 mL, time = 24 h, (n = 3). SF _REE/Metal_ is calculated using Equation (3).

Mixture	Metals (M)	Extraction (%)	SF _REE/Metal_
1	Nd	100	
	Co	0	Quant
	B	0	Quant
2	Sm	65 ± 5	
	Co	0	Quant
	Cu	0	Quant
3	La	100	
	Co	13 ± 1	6970 ± 640
	Ni	10 ± 1	8900 ± 1000
	Cu	16 ± 2	5400 ± 850

Quant: quantitative separation (>99%).

## Data Availability

The original contributions presented in the study are included in the article; further inquiries can be directed to the corresponding author.
